# GLIS2 Promotes Epithelial‐Mesenchymal Transition and Gastric Cancer Progression by Regulating BGN to Activate the Wnt/β‐Catenin Pathway

**DOI:** 10.1002/kjm2.70103

**Published:** 2025-09-12

**Authors:** Juan Yan, Ya‐Peng Deng

**Affiliations:** ^1^ The Infection Control Office Hunan Cancer Hospital Changsha Hunan Province People's Republic of China; ^2^ Endoscopy Center Hunan Cancer Hospital Changsha Hunan Province People's Republic of China

**Keywords:** BGN, epithelial‐mesenchymal transition, gastric cancer, GLIS2, progression

## Abstract

This study elucidates the mechanism by which GLIS Family Zinc Finger 2 (GLIS2) promotes epithelial‐mesenchymal transition (EMT) in gastric cancer through biglycan (BGN) activation and Wnt/β‐catenin stimulation. By analyzing 18 pairs of GC tissues and establishing in vitro models (combining GLIS2 knockdown/BGN overexpression with Wnt pathway modulators), we demonstrated that GLIS2 directly binds to the BGN promoter to enhance its transcription, thereby activating Wnt/β‐catenin signaling and significantly promoting GC cell migration, invasion, and EMT. Functional rescue experiments confirmed that BGN overexpression reverses the inhibitory effects of GLIS2 knockdown, while the Wnt/β‐catenin inhibitor XAV‐939 effectively blocks BGN's tumor‐promoting effects. These findings establish the crucial role of the GLIS2‐BGN‐Wnt/β‐catenin axis in regulating GC EMT and identify novel potential therapeutic targets for GC treatment.

**Abbreviations**
AKT: (alias PKB) protein kinase B
BGN: biglycan
CCK‐8: cell counting kit‐8
ChIP: chromatin immunoprecipitation
EdU: 5‐ethynyl‐2′‐deoxyuridine
EMT: epithelial‐mesenchymal transition
FBS: foetal bovine serum
GC: gastric cancer
GLIS2: GLIS family zinc finger 2
GTEx: genotype‐tissue expression
Ins2: Insulin 2
mTOR: mammalian target of rapamycin
MUT: mutant
PI3K: phosphoinositide 3‐kinase
STAT3: signal transducer and activator of transcription 3
STR: short tandem repeats
TCGA: the cancer genome atlas
TNKS: tankyrases
WT: widetype

## 1 Introduction

Gastric cancer (GC), the fifth most common digestive tract cancer worldwide, has a very high incidence and lethality, which seriously threatens people's lives and health [1]. There are more than one million new cases of GC worldwide each year, ranking fourth among all cancers in terms of deaths [2]. Studies have shown that the high mortality rate associated with GC is primarily due to the poor prognosis of patients with advanced GC [3]. GC progression is closely associated with epithelial‐mesenchymal transition (EMT), a hallmark event that promotes GC invasive potential and progression [4]. For example, ApoC‐II induces EMT to promote the peritoneal metastasis of GC via the PI3K/AKT/mTOR pathway [5]. Therefore, the targeted treatment of EMT in GC is crucial. In the future, we need to understand the mechanisms underlying EMT progression in GC to facilitate the development of therapeutic strategies.

GLIS Family Zinc Finger 2 (GLIS2) is a nuclear transcription factor encoding five C2H2‐type zinc finger structural domains [6]. It plays a transcriptional regulatory role in various diseases, including cancer. Wang et al. found that GLIS2 acts as a transcriptional activator of STAT3 by alleviating postoperative vascular dysfunction [7]. GLIS2 selectively regulates PUMA transcription to promote colorectal cancer [8]. In addition, circTAB2 inhibits lung cancer cell proliferation, migration, and invasion by regulating GLIS2 [9]. Although GLIS2 has been implicated in various malignancies, its precise functional mechanisms in GC pathogenesis, particularly in EMT regulation, remain unclear. Notably, one study demonstrated that GLIS2 expression is increased in GC and is associated with a poor prognosis [10]; yet the underlying molecular mechanisms remain to be elucidated.

It is widely known that the Wnt/β‐catenin pathway has a crucial effect on the EMT progression in cancer [11]. Cinobufacini inhibited colon cancer invasion and metastasis through inhibition of the Wnt/β‐catenin pathway and EMT [12]. Furthermore, several studies have shown that aberrant engagement of the Wnt/β‐catenin pathway drives EMT and GC progression. For example, SERPINH1 facilitated EMT and GC metastasis by enhancing the Wnt/β‐catenin pathway [13]. CCT5 induces EMT by activating the Wnt/β‐catenin pathway to promote GC lymph node metastasis [14]. Notably, GLIS2 regulates the EMT and apoptosis of renal tubular cells through the β‐catenin pathway in diabetic nephropathy [15]. Thus, we speculated that GLIS2 promoted the EMT process in GC through activation of the Wnt/β‐catenin pathway to exert a pro‐cancer effect.

Biglycan (BGN), an important component of extracellular matrix proteins, is a member of the family of leucine‐rich small‐molecule proteoglycans that play key roles in many organs and tissue systems [16]. In addition, several studies have found that the abnormal expression of BGN is linked to the prognosis, invasion, and metastasis of GC [17]. For example, the BGN/FAP/STAT3 positive feedback loop‐mediated interactions between the tumor and mesothelial cells contribute to GC peritoneal metastasis [18]. BGN is aberrantly upregulated in GC and is mainly associated with lymph node metastasis and depth of infiltration in GC [19]. BGN enhances GC invasion by activating the FAK signaling pathway [20]. Notably, BGN has been reported to be a key factor [21] in EMT in several cancers. LINC00460 promotes cell proliferation, migration, invasion, and EMT in squamous cell carcinoma of the head and neck by upregulating BGN [22]. Importantly, BGN can promote cancer metastasis by enhancing Wnt/β‐catenin [23]. Interestingly, in our study, using the GEPIA database, GLIS2 was found to have a positive relationship with BGN in GC, and the binding sites between GLIS2 and the BGN promoter were revealed using the JASPAR database. Based on these findings, we conjectured that GLIS2 affected the Wnt/β‐catenin pathway through activation of BGN transcription and thus promoted EMT in GC. This study reveals that GLIS2 may be a key upstream regulator of the Wnt/β‐catenin pathway, offering a new therapeutic target for GC treatment.

Taken together, we surmised that GLIS2 facilitated BGN transcription, which activated the Wnt/β‐catenin pathway and further promoted EMT, migration, and invasion in GC. This study investigated the molecular mechanisms by which GLIS2 regulates the EMT in GC. This study provides a new direction for the development of novel treatments for GC.

## 2 Materials and Methods

### 2.1 Patients With GC and Tissue Specimens

This study included 18 patients diagnosed with GC at Hunan Cancer Hospital (inclusion criteria were that they had not received systemic radiotherapy or chemotherapy). Clinicopathological characteristics, including tumor stage, histological grade, and metastasis status, were documented for all patients (see Table S1 for details). The table shows the correlation between GLIS2/BGN expression patterns and clinicopathological features, revealing that GLIS2‐high expression frequently coexists with high BGN levels and is associated with lymph node metastasis. Cancerous and noncancerous adjacent tissues from patients with GC were collected during surgery. Patients were briefed about the study and signed a written informed consent form prior to sample collection. The clinical samples used in this study were approved by the Ethics Committee of Hunan Cancer Hospital.

### 2.2 Cell Culture

The normal gastric epithelial cell line GES‐1 (SNL‐304) was purchased from Sunncell (Wuhan, Hubei, China). Human GC cell lines MKN74 (CL‐0730), MKN1 (CL‐0982), KATO‐III (CL‐0372), and AGS (CL‐0022) were purchased from Procell (Wuhan, Hubei, China). SNU‐16 (C6870) was purchased from Biotechnology (Shanghai, Songjiang, China). All cells were correctly identified using STR. The GES‐1, MKN74, MKN1, and SNU‐16 cell lines were cultured in RPMI‐1640 medium (11,875,093, Gibco, USA) supplemented with 1% penicillin/streptomycin (C0222, Biotech, China) and 10% FBS (10,099,141, Gibco, USA). KATO‐III cells were cultured in IMDM (PM150510; Procell, China) supplemented with 1% penicillin/streptomycin (P/S) and 10% FBS. AGS cells were grown in Ham's F‐12 medium (PM150810; Procell, China) supplemented with 1% P/S and 10% FBS. The cells were incubated at 5% CO_2_, 95% air, and 37°C. A 1:2 passage was performed when the cell lines reached 70%–80% density.

### 2.3 Cell Transfection and Treatment

The sh‐GLIS2, oe‐GLIS2, and oe‐BGN plasmids (GenePharma, Shanghai, China) were transfected into GC cells using Lipo 2000 (11,668,030, Invitrogen, USA) and cultured in Opti‐MEM (2,537,156, Gibco, USA) for 6 h. Subsequently, the medium was changed to the normal medium, and 10 μM of the Wnt/β‐catenin inhibitor XAV‐939 (S1180, Selleck, USA) or the Wnt/β‐catenin activator CHIR‐99021 (S1263, Selleck, USA) was added for 48 h [24]. All sh‐GLIS2 and overexpression constructs were validated for targeting efficiency in preliminary experiments using RT‐qPCR and western blotting (data shown in Figures S2 and 5). Detailed sequence information is provided in Table S2.

### 2.4 Western Blotting

The samples were lysed on ice for 10 min using RIPA solution (P0013B, Biotech, China) containing protease inhibitors (P1006, Biotech, China). Protein concentration was determined using a BCA kit (Biotech, P0011, China). Proteins were isolated in SDS‐PAGE gels and then transferred to PVDF membranes. The membranes were then cut and closed in 1% BSA for 2 h. Primary antibodies were hybridized at 4°C overnight such as GLIS2 (1:1000, Thermo Fisher, PA5‐72849, USA), E‐cadherin (1:1000, CST, #3195, USA), anti‐Claudin‐1 (1:1000, CST, #4933), ZEB1(1:1000, CST, #3396), Slug(1:1000, CST, #9585), Snail (1:1000, CST, #3879), WNT1(1:1000, Abcam, ab15251, USA), β‐catenin (1:1000, CST, #9562), p‐β‐catenin (1:1000, CST, #4176), c‐MYC (1:1000, CST, #9402), Cyclin D1 (1:1000, CST, #55506), and BGN (1:1000, Abcam, ab109369). The HRP‐conjugated anti‐rabbit IgG (1:2000, #7074, CST) was then introduced and maintained at 37°C for 1 h. A gel imaging system (1,708,195, Bio‐Rad, USA) was used to process the protein bands after incubation with ECL chromogenic solution (P0018S, Biotech, China).

### 2.5 RT‐qPCR

Cells or tissues (thoroughly ground) were lysed using Trizol, and then RNA was extracted using chloroform and isopropanol. The RNA was dissolved in 10 μL of DEPC, and the concentration was determined. Subsequently, the RNA was reverse‐transcribed into cDNA using a reverse transcription kit (RR037A, Takara, Japan). The qPCR premix was configured using the TB Green kit (RR420A, Takara) and assayed by the qPCR instrument (LightCycler96, Roche, Switzerland). The primer sequences used were as follows:

**Table:** 

h‐GLIS2‐F	CTGCCTCCTCCTTCCTTACC
h‐GLIS2‐R	CGGGCTTGACATGGTAATCG
h‐BGN‐F	CTCTGTCACACCCACCTACA
h‐BGN‐R	CGGAGATGTCGTTGTTCTGC
H‐β‐Actin‐F	CCCTGGAGAAGAGCTACGAG
H‐β‐Actin‐R	CGTACAGGTCTTTGCGGATG

### 2.6 CCK‐8

The experimental steps were performed according to the instructions of the CCK‐8 kit (C0039; Biotech, China). In each well, 10 μL of CCK‐8 solution was added and incubated for 1–4 h. Subsequently, the OD values at 450 nm were determined using an enzyme marker (Dynex MRX II, Spectra MR, USA).

### 2.7 5‐Ethynyl‐2′‐Deoxyuridine (EdU)

The EdU working solution was added to a 96‐well plate for 2 h. Next, 4% paraformaldehyde was added for 30 min, and 0.5% Triton X‐100 (P0096, Biotech, China) was incubated for 20 min. The reaction mixture was prepared according to the instructions of the BeyoClick EdU kit (C0071S; Biotech, China) and protected from light for 30 min. Finally, the nuclei were stained with DAPI (C1002, Biotech, China) and photographed to calculate the proliferating cell ratio.

### 2.8 Wound Healing Assay

GC cells (approximately 5 × 10^5^) were inoculated into 3 cm dishes and cultured until they adhered. When the cell density reached 100%, a 200 μL pipette tip drew a straight line across the cells. The cells were then rinsed with PBS. The cells were imaged after 0 and 24 h of incubation in serum‐free medium. The wound healing rate was calculated as healed area/wound area × 100%.

### 2.9 Transwell

Matrigel (C0372, Biotech, China) was pre‐coated into a Transwell chamber (FTW061, Biotech, China) and subsequently placed into a 24‐well plate for drying. Fresh medium containing FBS was added to the lower chamber, and a cell suspension was added to the upper chamber. The cells were incubated for 24 h in a cell culture incubator. The cells were then sequentially treated with 4% paraformaldehyde and 0.1% crystal violet (C0121; Biotech, China). The stained cells were photographed under a light microscope (IX73; Olympus, Japan).

### 2.10 Chromatin Immunoprecipitation (ChIP)

A ChIP kit (P2078, Beyotime, China) was used according to the manufacturer's instructions. Cell crosslinking was performed using 1% formaldehyde, and the cells were lysed by adding a cell lysis solution. Chromatin was fragmented by sonication into small fragments of 200–1,000 bp, which were then incubated with Protein A/G beads, GLIS2 antibody (1:100, GTX32148, GeneTex, USA), and control IgG for immunoprecipitation reactions to form Protein A/G bead‐GLIS2‐DNA complexes. Subsequently, the GLIS2‐DNA complexes were eluted from the magnetic beads and decross‐linked. Finally, the DNA was purified and assayed using agar gel electrophoresis to analyze the binding relationship between GLIS2 and BGN.

### 2.11 Dual Luciferase Reporter Gene Assay

Binding sites between GLIS2 and the BGN promoter were predicted using the JASPAR database. The WT or MUT gene sequences of the binding site were constructed from the predicted results and cloned into the pmirGLO vector (VT1439, Youbia, China). Subsequently, the constructed reporter plasmids were transfected into 293 T cells overexpressing GLIS2, and the cells were lysed after 48 h of culture. Next, dual luciferase activity was assessed using a dual luciferase reporter gene kit (RG027, Biotech, China) to analyze the binding relationship between GLIS2 and the BGN promoter.

### 2.12 Bioinformatics Analysis

This study conducted integrated bioinformatics analyses utilizing both the GEPIA (http://gepia.cancer‐pku.cn/) and JASPAR (http://jaspar.genereg.net/) databases. We extracted GLIS2 and BGN gene expression data from both the TCGA gastric cancer dataset and the GTEx normal tissue dataset using the GEPIA database. Pearson correlation analysis was employed to evaluate the association between GLIS2 and BGN expression levels, with |r| > 0.3 and *p* < 0.05 considered statistically significant. For transcription factor binding site prediction, the JASPAR database was used to identify potential GLIS2 binding sites within the BGN promoter region. The 2000 bp upstream sequence from the transcription start site of the BGN gene was defined as the promoter region, and potential transcription factor binding sites were screened using default parameters. Subsequently, enrichment analysis of the predicted binding sites was performed using online tools (e.g., the MAST tool integrated in JASPAR) to further evaluate their reliability and potential functional significance.

### 2.13 Statistical Analysis

Results are expressed as mean ± SD. Data were processed using Prism 9.0. A t‐test was used for comparisons between two groups. Multiple comparisons were performed using one‐way analysis of variance (ANOVA), followed by Tukey's post hoc test. *p* < 0.05 was deemed to indicate a statistically significant result.

## 3 Results

### 3.1 GLIS2 Expression Was Increased in GC and Related to Poor Prognosis

**FIGURE 1:**
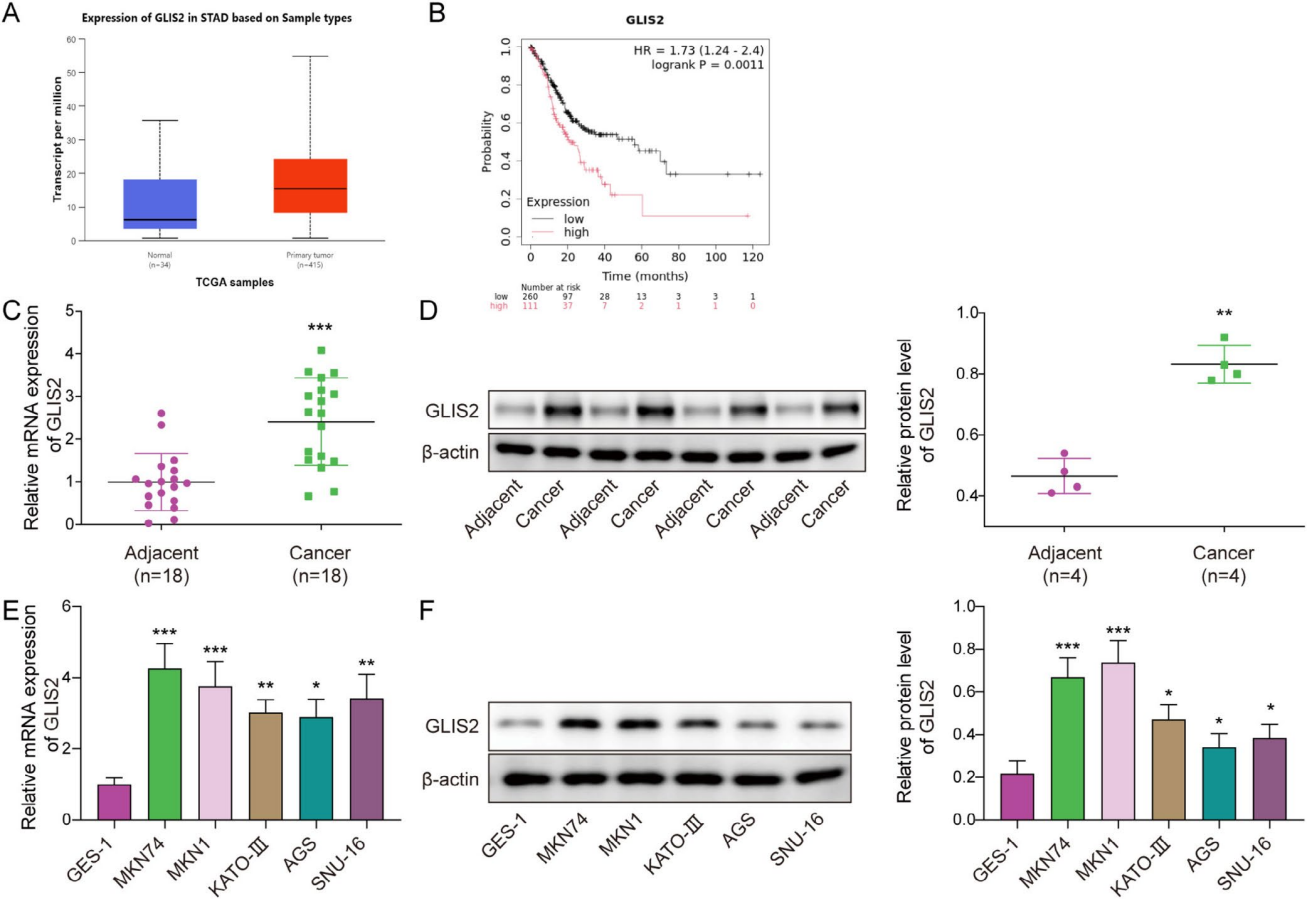
GLIS2 expression was increased in GC and related to poor prognosis. (A) The UALAN database was used to predict GLIS2 expression in GCs. (B) The association of GLIS2 with prognosis was analyzed by the Kaplan–Meier method. Eighteen pairs of cancerous and paraneoplastic tissues from patients with GC were collected for experimental analysis. (C) The mRNA expression of GLIS2 was detected using RT‐qPCR. *N* = 18. Statistical significance was determined by Student's t‐test: ***p < 0.001. (D) The levels of GLIS2 were examined by western blotting. *N* = 4. Statistical significance was determined by Student's t‐test: **p < 0.01. RNA and protein were collected from normal gastric epithelial cells (GES‐1) and GC cell lines (MKN74, MKN1, KATO‐III, AGS, and SNU‐16), respectively. (E) RT‐qPCR was performed to detect the GLIS2 expression. (F) Western blotting was employed to analyze the protein level of GLIS2. The experiment was performed in triplicate. Statistical significance was determined by Student's t‐test: *p < 0.05, **p < 0.01, and ***p < 0.001.

The UALAN database predicted that GLIS2 was upregulated in GC (Figure 1A); Kaplan–Meier survival analysis revealed that high GLIS2 expression was associated with poor prognosis (Figure 1B). Cancer tissues and tissues adjacent to GCs were collected, and GLIS2 was found to be significantly increased in GC tissues by western blotting and RT‐qPCR (Figure 1C,D). Experiments using GES‐1 as a control showed that GLIS2 was upregulated in GC cell lines (including MKN74, MKN1, KATO‐III, AGS, and SNU‐16), with the most significant changes in MKN74 and MKN1 (Figure 1E,F). These results indicated that GLIS2 may be a driver of GC.

### 3.2 GLIS2 Facilitated EMT, Migration, and Invasion of GC

**FIGURE 2:**
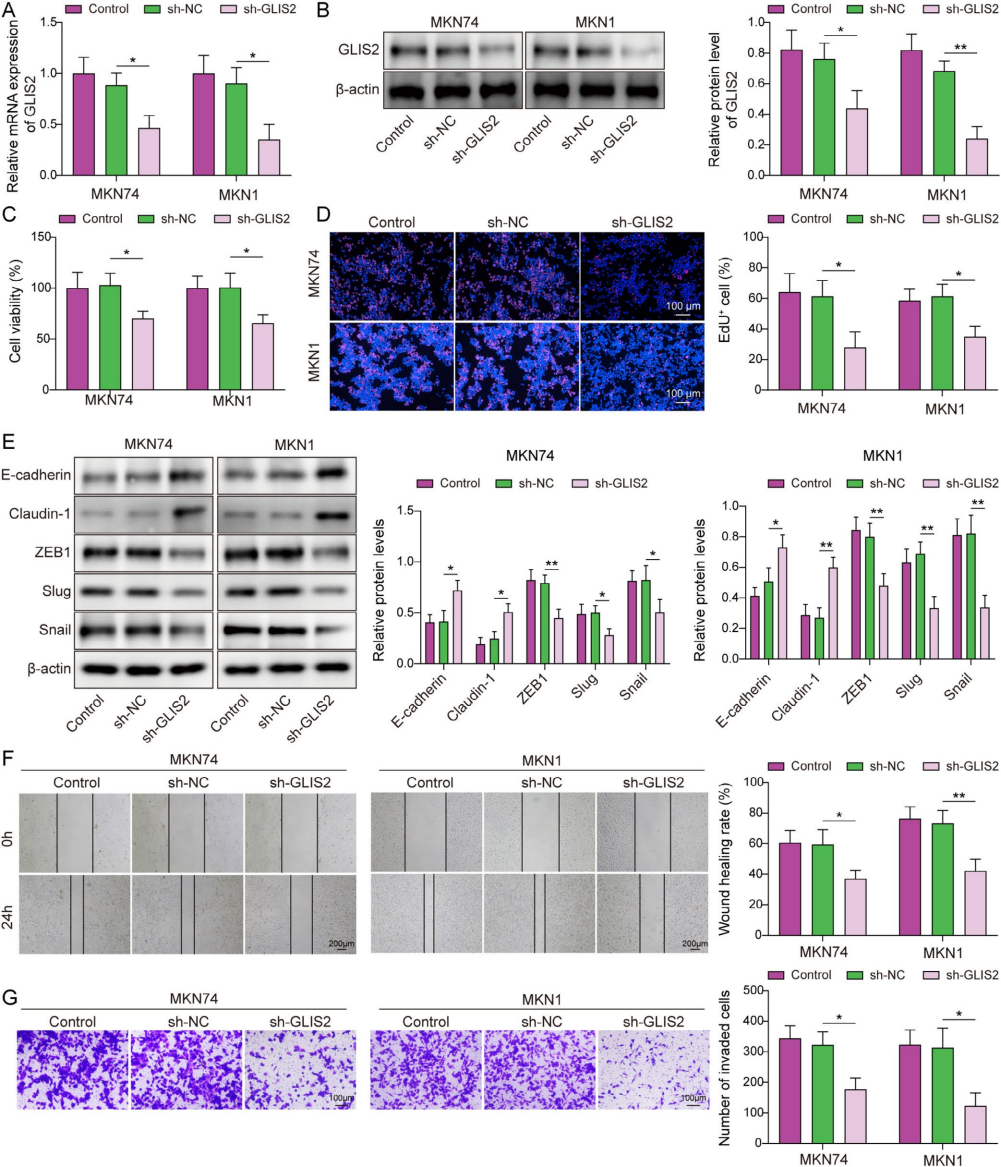
Knockdown of GLIS2 inhibited EMT, migration, and invasion of GC. (A) cellular model of GLIS2 knockdown was constructed using the MKN74 and MKN1 cells. (A, B) The knockdown efficiency of GLIS2 was analyzed using RT‐qPCR and western blotting. (C) The CCK‐8 assay was used to determine cell viability. (D) Cell proliferation was assessed by EdU staining. (E) The levels of E‐cadherin, Claudin‐1, ZEB1, Slug, and Snail were examined using western blotting. (F) A wound healing assay was employed to analyze cell migration ability. (G) Cell invasion was measured using a Transwell assay. The experiment was performed in triplicate. Statistical significance was determined using one‐way ANOVA followed by Tukey's post hoc test: *p < 0.05 and **p < 0.01.

Next, we constructed GLIS2 knockdown and overexpression cell models to investigate the role of GLIS2 in development and progression. After silencing GLIS2 in MKN74 and MKN1 cells, GLIS2 expression decreased (Figure 2A,B), whereas GLIS2 overexpression in AGS cells markedly increased its expression (Figure S1A,B). The experimental results demonstrated that GLIS2 knockdown reduced GC cell viability and proliferation (Figure 2C,D), whereas its overexpression exerted opposing effects (Figure S1C,D). Aberrant reactivation of EMT is critical for cancer cell invasive potential [25], featured by the deletion of epithelial cell markers (e.g., E‐cadherin and Claudin‐1) and upregulation of mesenchymal cell markers (e.g., ZEB1, Slug, and Snail) [26]. We observed that the levels of E‐cadherin and Claudin‐1 increased, the expression of ZEB1, Slug, and Snail was downregulated, and the EMT capacity was reduced after silencing GLIS2 (Figure 2E), with the ability of GC cells to migrate and invade (Figure 2F,G). Conversely, GLIS2 overexpression enhanced AGS cell migration, invasion, and EMT (Figure S1E–G). As a result, GLIS2 effectively promoted the migration, proliferation, invasion, and EMT of GC cells.

### 3.3 GLIS2 Promoted EMT, Migration, and Invasion of GC by Activating the Wnt/β‐Catenin Pathway

**FIGURE 3:**
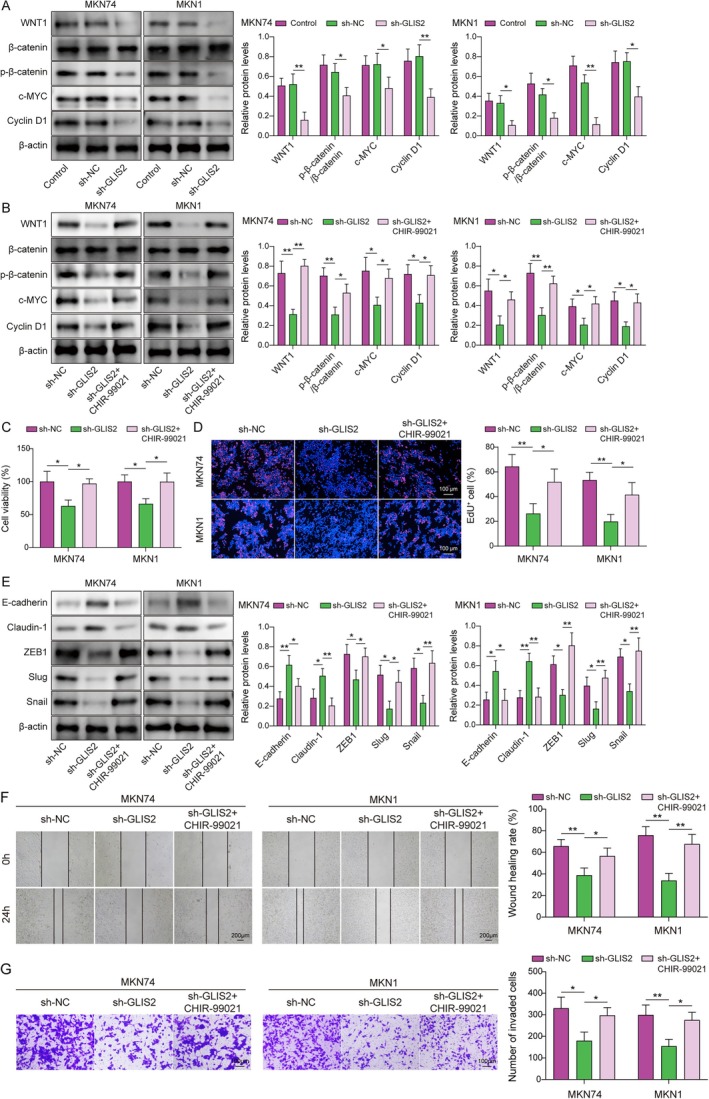
GLIS2 promoted EMT, migration, and invasion of GC by activating the Wnt/β‐catenin pathway. The selected GC cell lines MKN74 and MKN1 were used to silence GLIS2. (A) Western blotting was used to analyze the protein levels of WNT1, β‐catenin, p‐β‐catenin, c‐MYC, and Cyclin D1. Subsequently, a Wnt/β‐catenin activator (CHIR‐99021) was added. (B) The expression levels of WNT1, β‐catenin, p‐β‐catenin, c‐MYC, and Cyclin D1 were examined by western blotting. (C) CCK‐8 was used to assay the cell viability. (D) EdU was used to analyze cell proliferation. (E) The levels of E‐cadherin, Claudin‐1, ZEB1, Slug, and Snail were determined using western blotting. (F) Cell migration was determined using a wound healing assay. (G) Transwell was used to analyze cell invasion. The experiment was performed in triplicate. Statistical significance was determined using one‐way ANOVA followed by Tukey's post hoc test: *p < 0.05, **p < 0.01. [Correction added on 06 January 2026, after first online publication: Figure 3 has been replaced in this version.]

Immediately, to elucidate the potential molecular mechanisms by which GLIS2 regulates GC progression, we discovered that the expression of Wnt/β‐catenin pathway genes (e.g., WNT1, β‐catenin, p‐β‐catenin, c‐MYC, and Cyclin D1 [24]) was decreased in the constructed GLIS2‐silenced cell models (Figure 3A). Subsequently, to further investigate the effect of the Wnt/β‐catenin pathway on GC progression, we added 10 μM of Wnt/β‐catenin pathway activator (CHIR‐99021) to the GLIS2 knockdown GC cell model for experiments. As seen in Figure 3B, the results revealed that the levels of all the above Wnt/β‐catenin pathway factors were increased when the cells were treated with CHIR‐99021 for 24 h. In addition, CHIR‐99021 rescued the influence of silencing GLIS2 on the proliferation, migration, invasion, and expression of EMT markers in MKN74 and MKN1 cells (Figure 3C–G). The above data suggested that GLIS2 promoted GC progression by facilitating activation of the Wnt/β‐catenin pathway.

### 3.4 GLIS2 Facilitated the Transcription of BGN

**FIGURE 4:**
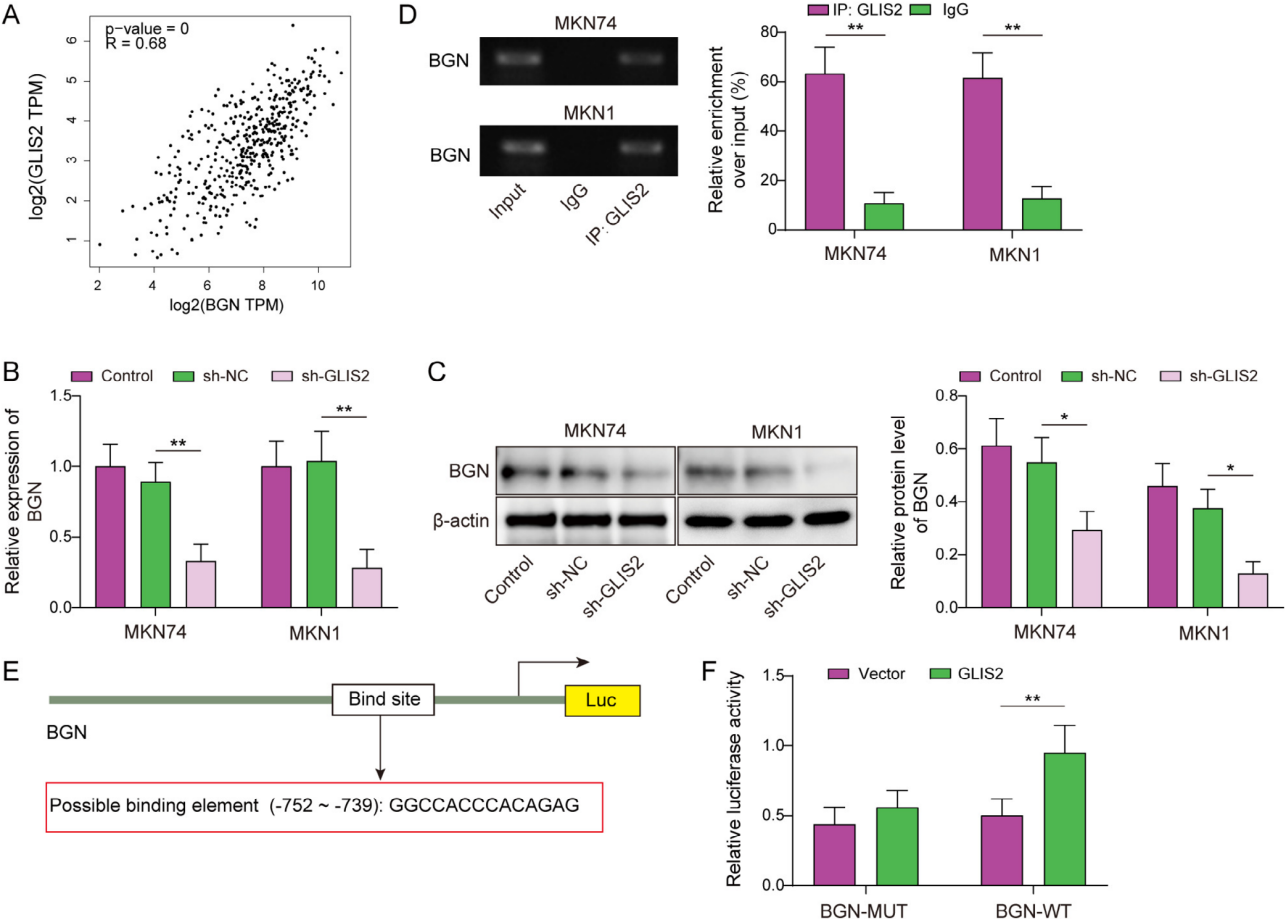
GLIS2 facilitated the transcription of BGN. (A) The GEPIA database was used to predict the relationship between BGN and GLIS2 in GC. (B, C) BGN expression by RT‐qPCR and western blotting after knockdown of GLIS2 in MKN74 and MKN1. (D) The binding association between GLIS2 and BGN was validated using ChIP in MKN74 and MKN1. (E) JASPAR was used to analyze the binding sites between GLIS2 and the BGN promoter. (F) The dual luciferase assay was employed to substantiate the combination of GLIS2 with BGN promoter. The experiment was performed in triplicate. Statistical significance was determined by Student's t‐test (two groups) or one‐way ANOVA followed by Tukey's post hoc test (multiple groups): *p < 0.05 and **p < 0.01.

Subsequently, we utilized the GEPIA database to obtain a positive correlation between BGN and GLIS2 in GC (Figure 4A). Moreover, BGN expression was downregulated by silencing GLIS2 in MKN74 and MKN1 cells (Figure 4B,C). ChIP experiments showed that GLIS2 was bound to the BGN promoter (Figure 4D). Then, we discovered that GLIS2 can bind to the BGN promoter using the JASPAR database (Figure 4E), which was verified by a dual luciferase reporter gene assay (Figure 4F). These data indicated that GLIS2 binds to the promoter region of BGN to promote its transcriptional activation, resulting in increased levels of BGN protein in GC.

### 3.5 GLIS2 Promoted EMT, Migration, and Invasion of GC by Increasing BGN to Activate the Wnt/β‐Catenin Signaling Pathway

**FIGURE 5:**
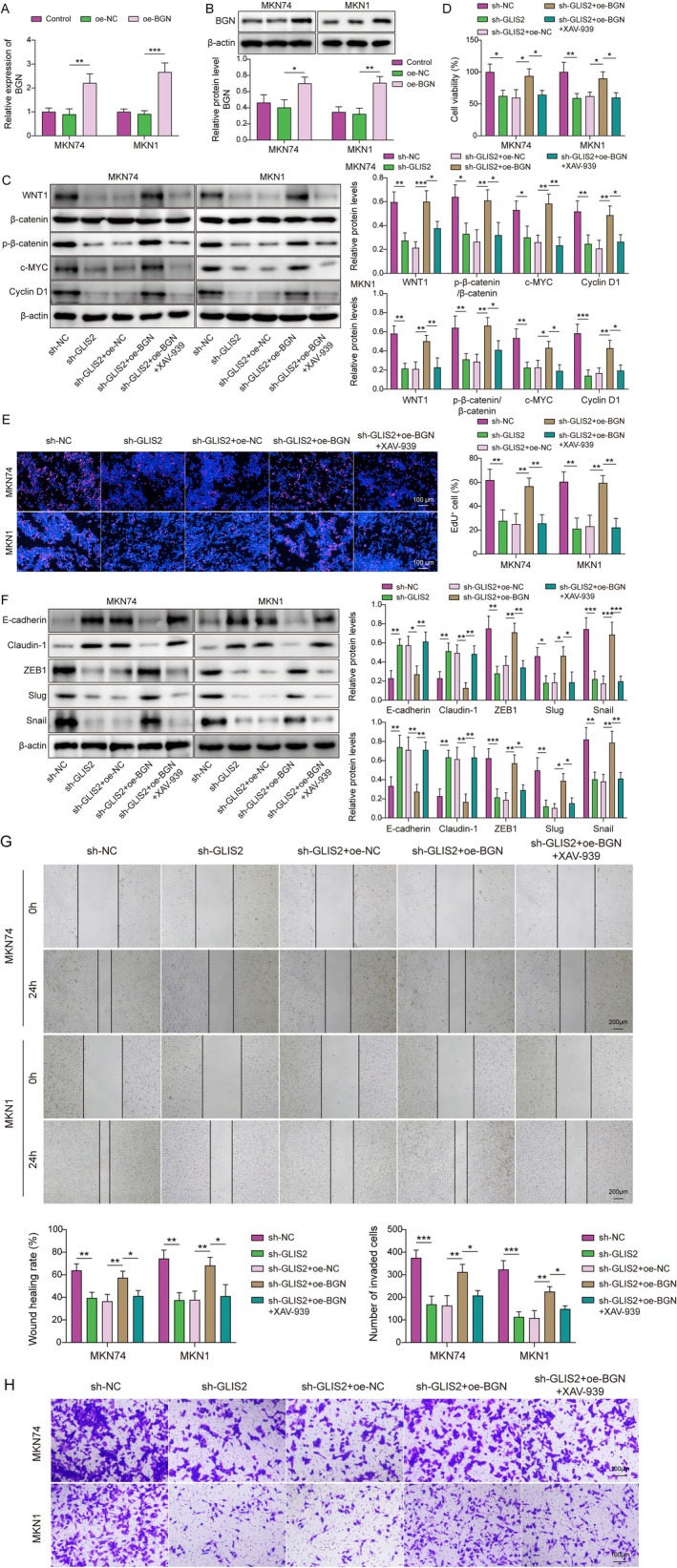
GLIS2 promoted EMT, migration, and invasion of GC by increasing BGN to activate the Wnt/β‐catenin signaling pathway. (A, B) MKN74 and MKN1 were selected to overexpress BGN, and increased BGN expression was verified by RT‐qPCR and western blotting. Silencing of GLIS2 with overexpression of BGN was followed by the addition of Wnt/β‐catenin signaling inhibitor (XAV‐939) for treatment in MKN74 and MKN1. (C) The levels of WNT1, β‐catenin, p‐β‐catenin, c‐MYC, and Cyclin D1 were examined using western blotting. (D) The cell viability was detected by CCK‐8. (E) EdU was used to analyze cell proliferation. (F) The expressions of E‐cadherin, Claudin‐1, ZEB1, Slug, and Snail were checked by western blotting. (G) Cell migration ability was determined using the wound healing assay. (H) Transwell was used to research cell invasion. The experiment was performed in triplicate. Statistical significance was determined by one‐way ANOVA followed by Tukey's post hoc test: *p < 0.05, **p < 0.01, and ***p < 0.001.

Finally, we silenced GLIS2 and overexpressed BGN (transfection efficiency as in Figure 5A,B) in GC cells, with added 10 μM of a Wnt/β‐catenin pathway inhibitor (XAV‐939). As in Figure 5C, BGN salvaged the inhibitory influence of GLIS2 knockdown on the Wnt/β‐catenin pathway, whereas XAV‐939 caused the opposite effect. Subsequently, we explored the function of the GLIS2/BGN/Wnt/β‐catenin axis in GC progression. The results indicated that high BGN expression facilitated GC cell proliferation, migration, invasion, and EMT after GLIS2 knockdown, whereas XAV‐939 effectively reversed these processes (Figure 5D–H). Altogether, the results of the above data indicated that GLIS2 activated the Wnt/β‐catenin signaling pathway through up‐regulation of BGN to facilitate GC progression.

## 4 Discussion

GC is one of the major malignant tumors worldwide and is the leading cause of cancer‐related deaths [1]. However, current treatment options for GC are limited, and patient survival rates are low [27]. According to previous studies, EMT is strongly associated with GC metastasis and progression [4]. In the present study, GLIS2 was upregulated in GC and promoted GC cell migration, proliferation, invasion, and EMT. Meanwhile, we proposed for the first time that GLIS2 could activate the Wnt/β‐catenin signaling pathway through upregulation of BGN to facilitate EMT and malignant progression in GC. This study provides new targets for GC therapeutic drugs.

GLIS2, a Glis family member encoding a member with five C2H2‐type zinc finger structural domains [28], exhibits complex and context‐dependent roles in cancer pathogenesis, functioning as both a tumor promoter and suppressor. While it has been demonstrated to suppress tumor growth in lung and liver cancers by modulating genes involved in cell survival and proliferation [9, 29], elevated GLIS2 expression is correlated with chemotherapy resistance and poor prognosis in GC [10]. Our findings are consistent with those of a previous report that showed significant upregulation of GLIS2 in GC tissues and cell lines. Importantly, the Wnt/β‐catenin pathway has been widely reported to facilitate EMT and malignant progression in GC. For instance, dihydroartemisinin repressed proliferation, migration, the Wnt/β‐catenin pathway, and EMT in GC via TNKS [30]. EHD3 promotes EMT and GC progression via the Wnt/β‐catenin pathway [31]. Our results also demonstrated that GLIS2 activated the Wnt/β‐catenin pathway to enhance GC cell proliferation, migration, invasion, and EMT progression.

To dig deeper into the specific mechanism of Wnt/β‐catenin pathway activation by GLIS2, we predicted that GLIS2 bound to the BGN promoter through the JASPAR database. BGN, a pivotal extracellular matrix component, regulates EMT in various malignancies via multiple mechanisms. Accumulating evidence has demonstrated that BGN not only directly modulates EMT markers [22] but also promotes EMT progression through TLR signaling [32] and by shaping a cancer‐associated fibroblast (CAF)‐like tumor microenvironment [33]. In GC, BGN expression closely correlates with prognosis, invasion, and metastasis [17]. BGN enhances GC invasion by activating the FAK signaling pathway [20]. Importantly, BGN expression was tightly regulated by canonical signaling pathways, including TGF‐β and Wnt/β‐catenin [34, 35]. Our study provided novel insights by demonstrating for the first time that GLIS2 directly bound to the BGN promoter and significantly enhanced its transcriptional activity, with BGN expression showing a strong positive correlation with GLIS2 levels, thereby activating the GLIS2‐BGN‐Wnt/β‐catenin signaling axis to drive GC progression.

The present study revealed that GLIS2 activates the BGN/Wnt/β‐catenin pathway in GC, which contrasts with previous reports showing its inhibitory effect on β‐catenin signaling in diabetic nephropathy models [15]. This tissue‐specific functional divergence may stem from the dual functionality of GLIS2 as a nuclear transcription factor [28]. In the renal system, GLIS2 maintained the renal tubular epithelial phenotype by repressing Snai1 and Wnt4 promoter activity [36], while another study has shown that GLIS2 can function as a transcriptional activator by directly binding to the Ins2 promoter to enhance its expression in renal tubule epithelial cells [37]. The underlying mechanism for this functional diversity lies in the context‐dependent actions of GLIS2. Rather than functioning independently, GLIS2 dynamically interacts with tissue‐specific coregulators to form distinct transcriptional complexes, exhibiting cell type‐specific regulatory characteristics as demonstrated in previous studies [38]. For instance, research has shown that in hematopoietic stem cells, GLIS2 forms complexes with HDAC3 to maintain PPAR γ signaling activity [39], whereas in endothelial cells it promotes proliferation through STAT3 interaction [7]. The different degrees of repression and transactivation observed in the cell lines may be due to different levels of expression of co‐activators and co‐repressors able to interact with GLIS2 [38]. Additionally, post‐translational modifications of GLIS2, particularly the competitive regulation between SUMOylation and ubiquitination, significantly influence its stability and transcriptional activity [40]. Based on these findings, we hypothesized that gastric cancer cells might express specific transcriptional cofactors cooperating with GLIS2 to activate BGN expression. This hypothesis requires further experimental validation.

In summary, this work suggested that GLIS2 increased BGN transcription to activate the Wnt/β‐catenin pathway, driving the migration, proliferation, invasion, and EMT progression of GC cells. It can be reasonably deduced that the inhibition of GLIS2 may be a promising approach for the treatment of GC. Moreover, this study elucidates the critical role of the GLIS2‐BGN‐Wnt/β‐catenin signaling axis in GC EMT progression, providing novel insights for clinical management. As prognostic biomarkers, the co‐expression patterns of GLIS2 and BGN correlated with tumor invasion depth, lymph node metastasis, and clinical staging, suggesting their potential as novel molecular signatures for EMT‐type GC, particularly for metastatic risk assessment and personalized prognosis prediction. Therapeutically, the cascade regulatory characteristics of this axis offer opportunities for multilevel intervention. For example, small‐molecule inhibitors targeting the GLIS2‐DNA binding domain may block BGN transcriptional activation. BGN monoclonal antibodies can inhibit their interaction with the tumor microenvironment, and their combination with inhibitors of the Wnt/β‐catenin pathway may yield synergistic antitumor effects.

However, several important limitations of this study must be acknowledged. First, while a small cohort of 18 clinical samples showed co‐occurrence of elevated GLIS2 expression with increased BGN levels and was associated with lymph node metastasis, the relatively limited sample size resulted in some non‐significant *p*‐values. Secondly, due to limitations in experimental conditions, investigations into in vivo experiments are lacking. Future studies should expand the sample size to validate clinical relevance, establish humanized mouse models to evaluate targeted therapeutic effects, and employ single‐cell sequencing to analyze the specific regulatory networks of this axis across different molecular subtypes of GC, thereby providing more robust evidence‐based support for developing precision diagnosis and treatment strategies.

## Ethics Statement

Patients were briefed about the study and signed a written informed consent form before collecting samples. The clinical samples involved in this study were approved by the Ethics Committee of Hunan Cancer Hospital.

## Conflicts of Interest

The authors declare no conflicts of interest.

## Supporting information


**Figure S1:** Effects of GLIS2 overexpression on AGS cells. (A, B) The overexpression efficiency was validated by RT‐qPCR and Western blot. (C) Cell viability was assessed by CCK‐8 assay. (D) Cell proliferation was evaluated using EdU assay. (E) The expressions of E‐cadherin, Claudin‐1, ZEB1, Slug, and Snail were tested using Western blot. (F) Cell migration was examined by wound healing assay. (G) Cell invasion was measured via Transwell assay.
**Figure S2:** Validation of GLIS2 knockdown efficiency. (A) GLIS2 mRNA levels were detected by RT‐qPCR. (B) The expression of GLIS2 was examined using Western blot analysis.
**Table S1:** Patient clinical information.
**Table S2:** The primers for knock‐in expression and overexpression.

## Data Availability

Data sharing not applicable to this article as no datasets were generated or analysed during the current study.
