# Supplementary material for: GLIS2 Promotes Epithelial‐Mesenchymal Transition and Gastric Cancer Progression by Regulating BGN to Activate the Wnt/β‐Catenin Pathway
Source: Kaohsiung J Med Sci. 2025 Sep 12;42(2):e70103. doi: 10.1002/kjm2.70103 (PMC12884764; doi:10.1002/kjm2.70103)
Supplement: Supplementary file 1 — Figure S1: Effects of GLIS2 overexpression on AGS cells. (A, B) The overexpression efficiency was validated by RT‐qPCR and Western blot. (C) Cell viability was assessed by CCK‐8 assay. (D) Cell proliferation was evaluated using EdU assay. (E) The expressions of E‐cadherin, Claudin‐1, ZEB1, Slug, and Snail were tested using Western blot. (F) Cell migration was examined by wound healing assay. (G) Cell invasion was measured via Transwell assay. Figure S2: Validation of GLIS2 knockdown efficiency. (A) GLIS2 mRNA levels were detected by RT‐qPCR. (B) The expression of GLIS2 was examined using Western blot analysis. Table S1: Patient clinical information. Table S2: The primers for knock‐in expression and overexpression. [file KJM2-42-e70103-s001.docx]

**Supplementary Materials**


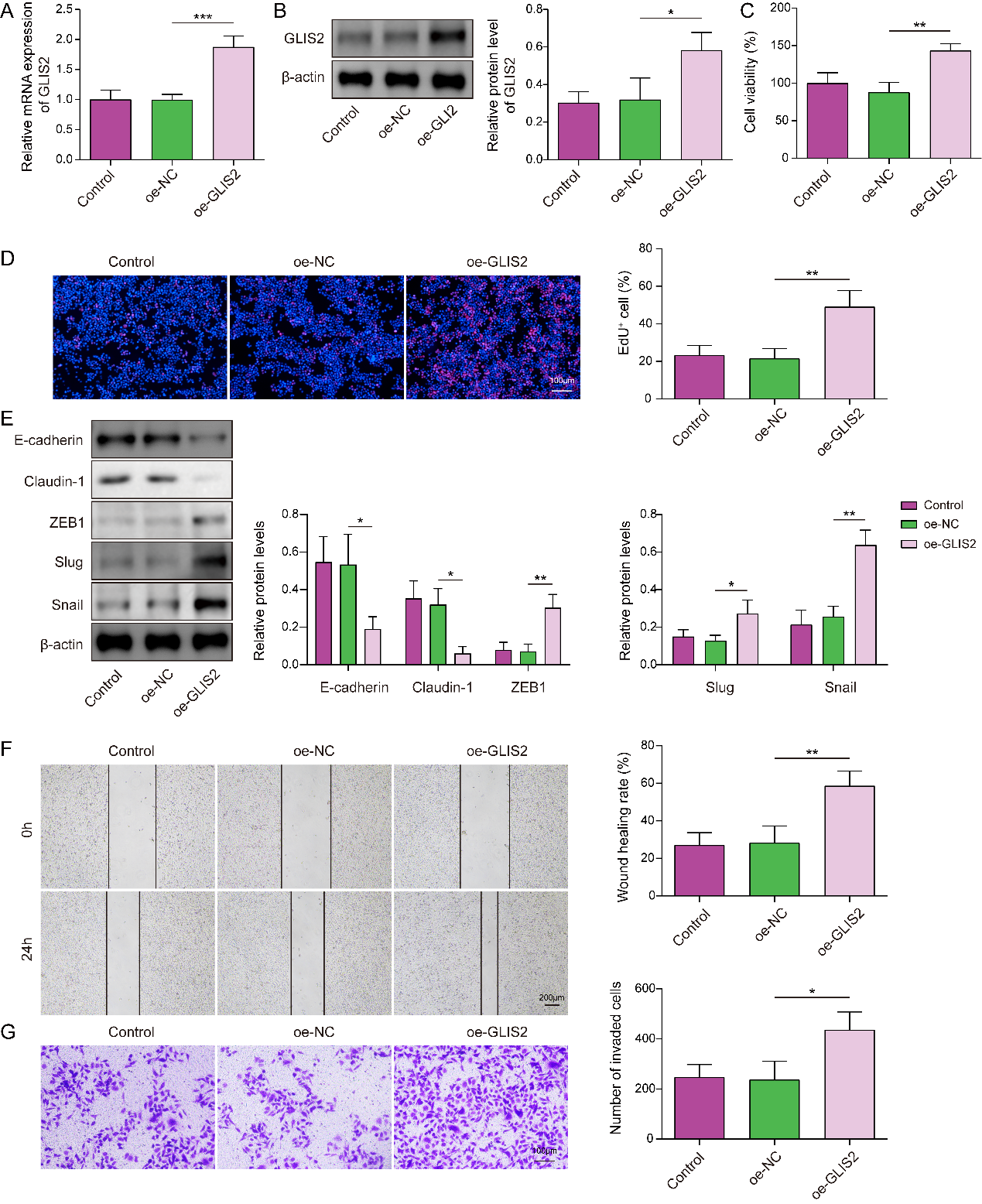


Figure S1. Effects of GLIS2 overexpression on AGS cells. A-B. The overexpression efficiency was validated by RT-qPCR and Western blot. C. Cell viability was assessed by CCK-8 assay. D. Cell proliferation was evaluated using EdU assay. E. The expressions of E-cadherin, Claudin-1, ZEB1, Slug, and Snail were tested using Western blot. F. Cell migration was examined by wound healing assay. G. Cell invasion was measured via Transwell assay.


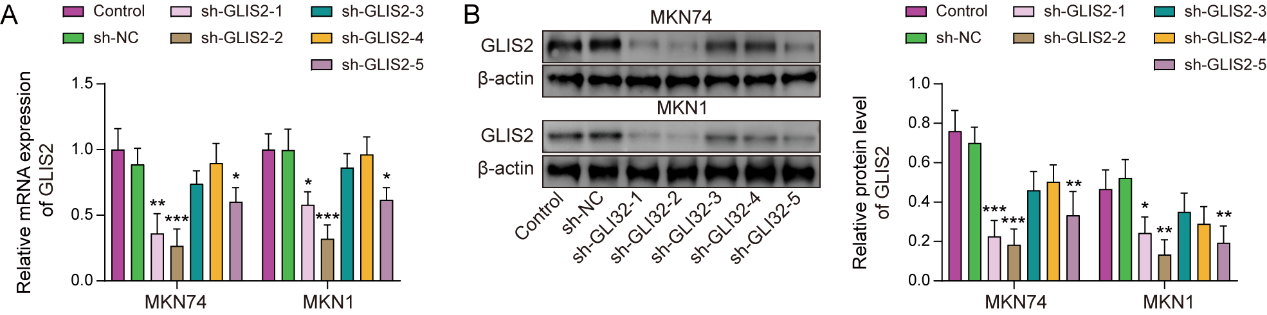


Figure S2. Validation of GLIS2 knockdown efficiency. A. GLIS2 mRNA levels were detected by RT-qPCR. B. The expression of GLIS2 was examined using Western blot analysis.

**Table S1： Patient clinical information**

| Clinicopathological Characteristics | GLIS2 | | | *P* | BGN | | | *P* |
| --- | --- | --- | --- | --- | --- | --- | --- | --- |
|  | N=18 | High (n=9) | Low (n=9) |  | N=18 | High (n=9) | Low (n=9) |  |
| **Gender** |  |  |  | *0.6372* |  |  |  | ***0.0498**** |
| Male |  | 5 | 3 |  |  | 8 | 3 |  |
| Female |  | 4 | 6 |  |  | 1 | 6 |  |
| **Age (years)** |  |  |  | *0.3348* |  |  |  | *0.6372* |
| < 60 |  | 4 | 7 |  |  | 3 | 5 |  |
| ≥ 60 |  | 5 | 2 |  |  | 6 | 4 |  |
| **TNM stage** |  |  |  | ***0.0498**** |  |  |  | ***0.0152**** |
| I–II |  | 3 | 8 |  |  | 2 | 8 |  |
| Ⅲ-IV |  | 6 | 1 |  |  | 7 | 1 |  |
| **Lymph node metastasis** |  |  |  | *0.6372* |  |  |  | *0.1534* |
| Negative |  | 3 | 5 |  |  | 2 | 6 |  |
| positive |  | 6 | 4 |  |  | 7 | 3 |  |
| **Distant metastasis** |  |  |  | *0.3469* |  |  |  | *0.3348* |
| Negative |  | 3 | 6 |  |  | 2 | 5 |  |
| positive |  | 6 | 3 |  |  | 7 | 4 |  |
| **Differentiation** |  |  |  | ***0.0152**** |  |  |  | *0.2941* |
| Well/ Moderate |  | 2 | 8 |  |  | 1 | 4 |  |
| poor |  | 7 | 1 |  |  | 8 | 5 |  |

The correlation between gene expression and clinicopathological parameters was analyzed using Fisher's exact test, with a p-value <0.05 considered statistically significant. The expression levels of GLIS2 and BGN were dichotomized based on the median value of all samples. Values ≥ the median were defined as high expression, while values < the median were defined as low expression.

**Table S2: The primers for knock-in expression and overexpression**

| **Gene Symbol** | | **sequence** |
| --- | --- | --- |
| oe-GLIS2  (5’-3’) | F: CAAGCTGGCTAGCGTTTAAACTTAAGCTTatgcactccctggacgagcc | |
|  | R: GCCACTGTGCTGGATATCTGCAGAATTCtcagttcaccacagccggtttg | |
| oe-BGN  (5’-3’) | F: CAAGCTGGCTAGCGTTTAAACTTAAGCTTatgtggcccctgtggcgc | |
|  | R: CACTGTGCTGGATATCTGCAGAATTCctactttttgtagttgccaaactggatgg | |
| sh-GLIS2-1 | CCGAGGTTTCAACGCCAGGTA | |
| sh-GLIS2-2 | | CCGCACACACACCAACGAGAA |
| sh-GLIS2-3 | | GACCATGTCAACGATTACCAT |
| sh-GLIS2-4 | | GCCTCTCAATCTGGCCAAGAA |
| sh-GLIS2-5 | | ATGTGGACAAGCCCTACTACT |
| sh-NC | | TTCTCCGAACGTGTCACGT |
